# Transcription of TIR1-Controlled Genes Can be Regulated within 10 Min by an Auxin-Induced Process. Can TIR1 be the Receptor?

**DOI:** 10.3389/fpls.2016.00995

**Published:** 2016-07-11

**Authors:** Corinna Labusch, Yunus Effendi, Martin Fulda, Günther F. E. Scherer

**Affiliations:** ^1^Abteilung Molekulare Ertragsphysiologie, Institut für Gartenbauliche Produktionssysteme, Leibniz Universität HannoverHannover, Germany; ^2^Department of Biology, University of Al Azhar IndonesiaJakarta, Indonesia; ^3^Abteilung Biochemie der Pflanzen, Albrecht-von-Haller-Institut der Pflanzenwissenschaften, Universität GöttingenGöttingen, Germany

**Keywords:** AUXIN-BINDING PROTEIN 1, early auxin-induced genes, auxin transport mutants, auxin receptor mutants, fatty acid-metabolism mutants, protein kinase mutants, protein phosphatase mutants, TRANSPORT INHIBITOR RESPONSE 1

## Abstract

ABP1 and TIR1/AFBs are known as auxin receptors. ABP1 is linked to auxin responses several of which are faster than 10 min. TIR1 regulates auxin-induced transcription of early auxin genes also within minutes. We use transcription of such TIR1-dependent genes as indicator of TIR1 activity to show the rapid regulation of TIR1 by exogenous auxin. To this end, we used quantification of transcription of a set of fifteen early auxin-induced reporter genes at *t* = 10 and *t* = 30 min to measure this as a TIR1-dependent auxin response. We conducted this study in 22 mutants of auxin transporters (*pin5, abcb1, abcb19, and aux1/lax3*), protein kinases and phosphatases (*ibr5, npr1, cpk3, CPK3-OX, d6pk1, d6pkl1-1, d6pkl3-2, d6pkl1-1/d6pkl2-2, and d6pkl1-1/d6pkl3-2*), of fatty acid metabolism (*fad2-1, fad6-1, ssi2, lacs4, lacs9, and lacs4/lacs9*) and receptors (*tir1, tir1/afb2, and tir1/afb3*) and compared them to the wild type. After 10 min auxin application, in 18 out of 22 mutants mis-regulated expression of at least one reporter was found, and in 15 mutants transcription of two-to-three out of five selected auxin reporter genes was mis-regulated. After 30 min of auxin application to mutant plants, mis-regulation of reporter genes ranged from one to 13 out of 15 tested reporter genes. Those genes chosen as mutants were themselves not regulated in their expression by auxin for at least 1 h, excluding an influence of TIR1/AFBs on their transcription. The expression of *TIR1/AFB* genes was also not modulated by auxin for up to 3 h. Together, this excludes a feedback or feedforward of these mutant genes/proteins on TIR1/AFBs output of transcription in this auxin-induced response. However, an auxin-induced response needed an as yet unknown auxin receptor. We suggest that the auxin receptor necessary for the fast auxin-induced transcription modulation could be, instead, ABP1. The alternative hypothesis would be that auxin-induced expression of a protein, initiated by TIR1/AFBs receptors, could initiate these responses and that this unknown protein regulated TIR1/AFB activities within 10 min.

## Introduction

Auxin plays an essential role in plant growth and development. Auxin is perceived by three receptors, ABP1 on the outer surface of the plasma membrane and the TIR1/AFBs in the nucleus (Badescu and Napier, 2006). The TIR1/AFB receptors are encoded by a small gene family (Parry et al., 2009); ABP1 is encoded by a single gene (Chen et al., 2001). A third auxin receptor, SKP2A, has a specialized function in activating the cell cycle (Jurado et al., 2010) and is not relevant for this study on auxin-induced transcription.

The mechanism of auxin binding to TIR1 and Aux/IAA proteins (auxin/indole-acetic acid proteins) in a sandwich complex has been investigated in great detail (Tan et al., 2007; Calderon-Villalobos et al., 2012; Salehin et al., 2015). Ubiquitination of the bound Aux/IAA proteins by SCF^TIR1^ and their subsequent proteolysis in the proteasome prevents them from binding to ARF transcription factors, thereby modulating the expression of early auxin-induced genes (Mockaitis and Estelle, 2008). TIR1/AFB activities, measured as degradation of IAA-luciferase proteins, was shown to increase within less than 2 min upon auxin administration (Zenser et al., 2003). A second method to measure TIR1 activity is the quantification of transcription of the early auxin-induced genes, which can thus be used as a specific test for TIR1/AFB activities (Salehin et al., 2015). Auxin-induced transcriptional changes can be measured within a few minutes (Abel and Theologis, 1996). Hence, transcription of auxin-inducible genes can be used like many other auxin biotests but with the great advantage that molecular details are already well-known and the chain of events from receptor to transcription control is short.

ABP1 is a small glycoprotein localized on the extracytosolic side of the plasma membrane and in the ER (Napier et al., 2002) and is important for many rapid cellular changes (Badescu and Napier, 2006). Recently it was shown that ABP1 plays no role in embryo development as previously postulated (Chen et al., 2001) so that its role in auxin signaling has been questioned (Dai et al., 2015; Gao et al., 2015). However, ABP1 binds to TMK1, a transmembrane receptor kinase, and this activates small G-protein signaling in the cytosol (Dai et al., 2013; Xu et al., 2014) and, conceivably, this mechanism is suitable to also activate other processes in the cytosol. Therefore, the transmembrane signaling function of ABP1 could potentially reach many targets within minutes.

It is more difficult to relate the TIR1/AFB receptors to rapid auxin responses within 5–10 min because transcription/translation is the mechanism of action of TIR1/AFBs, and regulation of proteins other than those translated from early auxin genes has not been proven to be so rapid. But the genes of numerous mutants in auxin signaling or physiology were described which are not part of the machinery regulating promoter activity of primary auxin genes.

The mutants used in this investigation and previously (Effendi et al., 2011, 2013, 2015; Labusch et al., 2013) are potentially related to rapid auxin responses. The transcription of all these mutant genes is not regulated by auxin during the first hour as was documented (Nemhauser et al., 2006^1^). Therefore, it is difficult to assume that there is transcriptional feedback or feedforward to TIR1/AFB activity through these mutated proteins/genes investigated here. But, we could find in the mutants used here and previously, changes in TIR1/AFB-controlled and auxin-induced transcription of early auxin-induced genes at *t* = 10 min and *t* = 30 min, thus indicating an auxin receptor-driven process influencing TIR1/AFB activities. This auxin receptor-driven process potentially acts in parallel or in addition to TIR1/AFBs receptors.

Known rapid auxin-induced responses measured within 5–10 min or even less influenced selection of the mutants in our investigations. Examples of rapid responses are the activation of the pPLA (Paul et al., 1998) and the H^+^-ATPase (Takahashi et al., 2012; Fuglsang et al., 2014). Both enzymes are phosphorylated and, additionally, auxin-induced phosphorylation was reported in other systems (Mockaitis and Howell, 2000) so that, we included auxinic mutants affecting protein phosphatases (*ibr5, npr1*) (Garbers et al., 1996; Monroe-Augustus et al., 2003) and kinases (*cpk3, CPK3-OX, d6pk1, d6pkl1-1, d6pkl3-2, d6pkl1-1/d6pkl2-2, and d6pkl1-1/d6pkl3-2*) (Zegzouti et al., 2006; Strader et al., 2008; Zourelidou et al., 2009, 2014; Mehlmer et al., 2010; Rietz et al., 2010). Furthermore, inhibition of clathrin-dependent endocytosis of PIN proteins is a rapid auxin response (Robert et al., 2010; Xu et al., 2010), as is auxin transport itself (Petrasek et al., 2006). This prompted us to include mutants in auxin transport proteins, PIN5 (Mravec et al., 2009) and previously PIN2 (Effendi and Scherer, 2011) as well as in ABCB1, ABCB19, and AUX1 (Geisler et al., 2003; Péret et al., 2012). Lipids are substrates for pPLA and PIN proteins interact with lipids (Roudier et al., 2010; Markham et al., 2011) both of which could influence the responses to auxin in mutants in membrane lipid metabolism. Mutants in *TIR1* and *AFB* genes were chosen because, so far, we had investigated only mutants of the *ABP1* gene.

As a method, we had previously used a set of transcripts (*IAA2, IAA3, IAA11, IAA13, IAA14, IAA19, IAA20, SAUR9, SAUR15, SAUR23, GH3-5, PIN1, PIN2, PIN3, and PIN5*) to report transcription induced by auxin and, hence, TIR1/AFB activities (Effendi et al., 2011, 2013, 2015; Labusch et al., 2013). Some are well-accepted rapid auxin response transcripts, like *SAURs*, and most belong to the known gene families of early auxin-induced genes (Vieten et al., 2005; Paponov et al., 2008).

As with the previously investigated mutants, our results support the observations that TIR1/AFB activities can be regulated within 10 min by an auxin-initiated and receptor-driven process. Despite some recent doubts about the signaling function of ABP1 (Gao et al., 2015), new findings on ABP1 indicate that ABP1 (Dai et al., 2013; Xu et al., 2014) could be an auxin receptor acting rapidly by a post-translational mechanism that can operate on a time scale from 0 to 10 min. This poses the question: can TIR1/AFBs be the receptors to initiate this response or is it more likely that a second receptor can fulfill such a function?

## Materials and Methods

### Plant Material and Growth Conditions

Seedlings seeds were surface- sterilized, stratified for 2–4 days, and grown under long day conditions (22°C; 16 h white light, 8 h dark, 30–40 μE) in 500 μL MS/2 liquid medium for 7 days in small hydrophilic Petri dishes (5 cm) until they had 2–4 primary leaves. Prior to treatment with auxin the medium was replaced by 450 μL fresh medium. After 4 h equilibration to the fresh medium, seedlings were treated either with 50 μL 100 μM IAA (10 μM final concentration) prepared from a 20 mM stock solution in DMSO or only with MS/2 liquid medium containing the same amount of DMSO for 10 or 30 min. Plant material was quickly blotted on filter paper and frozen in liquid nitrogen.

### Mutants Used in this Study

For quantification of TIR1-dependent transcription, we selected a set of reporter genes and included in addition several PIN genes of potential importance (*IAA2, IAA3, IAA11, IAA13, IAA14, IAA19, IAA20, SAUR9, SAUR15, SAUR23, GH3-5, PIN1, PIN2, PIN3, and PIN5*). The primary auxin responsive genes include three gene families called *Aux/IAA*, *GH3*, and *SAUR* (Abel and Theologis, 1996; Paponov et al., 2008). Expression of many of these genes was up-regulated within minutes of exposure to auxin and was independent of *de novo* protein synthesis (Abel and Theologis, 1996). Aux/IAA proteins are short lived and they play a crucial role in auxin-mediated signaling (Mockaitis and Estelle, 2008). The *GH3* gene family in *Arabidopsis* encodes IAA-amido synthetases that have the function to maintain IAA homeostasis in converting auxin to inactive amino acid conjugates (Staswick et al., 2005). Expression of *SAUR* mRNAs was induced by auxin within 2 to 5 min (Abel and Theologis, 1996). The protein function is still mostly unknown but they are thought to be involved in auxin signal transduction, auxin transport and elongation (Chae et al., 2012; Spartz et al., 2012, 2014). We also chose several additional genes of interest (*PIN1, PIN2, PIN3, and PIN5*), which we had used before to characterize *abp1* and *ppla* mutants (Effendi et al., 2011; Labusch et al., 2013). Details of the sources of the mutants are found in **Supplementary Table S1**.

### Nucleic Acid Analysis

For quantitative RT-PCR, we employed methods as previously described (Labusch et al., 2013). Total RNA from auxin treated seedlings was prepared using TRIzol reagent according to the manufacturer’s instructions (Invitrogen), treated with DNase I (Invitrogen) and converted to cDNA with RevertAid H Minus First Strand cDNA Synthesis kit (Fermentas). Primer efficiency was checked by using different cDNA concentrations and only primer with mathematical efficiency between 95 and 105% were used. Primers are listed in supplemental material (**Supplementary Table S1**). For quantitative PCR reactions SYBR-Green Master Mix was used in a StepOnePlus system (Applied Biosystem). About 30 ng cDNA, 200 nM primers, 0.5 μM ROX (Invitrogen), 0.1x SYBR Green (Invitrogen), and 0.03 U Hot Start Polymerase (DNA cloning service) were utilized in one PCR reaction. The specificity of PCR amplification was examined by monitoring the presence of a single peak in the melting curves for quantitative PCR. In each experiment four to six biological repeats, and for each biological treatment three technical repeats were performed for the subsequent qPCR reaction. Relative expression calculation and statistical analysis were done with REST 2009 software (Pfaffl et al., 2002). The values for *t* = 0 min in untreated wt and mutants were separately calculated relative to the *ubiquitin10* gene reference gene. The expression level of the untreated controls in the wt and the mutants was set as one fold and auxin modulation accordingly at *t* = 10 and *t* = 30 min values. Data on primers and sources of mutants are in **Supplementary Tables S1** and **S2**.

## Results

### Auxin Transport Mutants Modulate Expression of Reporter Genes

**FIGURE 1:**
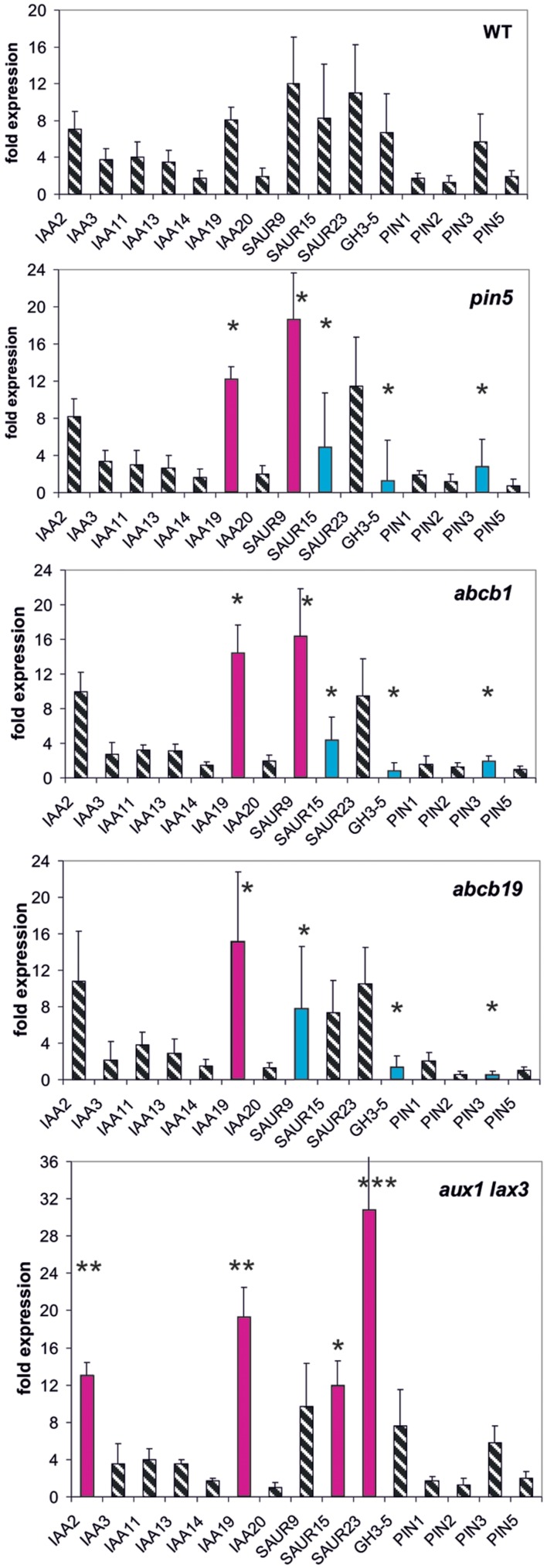
**Auxin-induced transcription of reporter genes in seedlings of mutants *pin5, abcb1, abcb19, aux1/lax3*, and wild type (wt) seedlings.** Seedlings were grown in the light for 7 days and auxin treatment was for 30 min. For each, the wt and the mutants, one panel is shown for the reporter genes. Red bars indicate significant increased and blue bars significantly decreased transcription of reporter genes in comparison between the wt and mutant. Striped bars indicate no significant difference between the wt and the mutant. Transcription in the untreated wt and mutants at *t* = 0 min were set at 1, and values after 10 μM treatment by IAA at *t* = 30 min were calculated relative to these values and represent as a bar either in the wt or in a mutant diagram [^∗^*p* < 0.05, ^∗∗^*p* < 0.01, ^∗∗∗^*p* < 0.001; *t*-test

Mutants of three different types of auxin transport proteins, *pin5, abcb1, abcb19* (efflux transporter), and the double mutant *aux1/lax3* (influx transporter) (Geisler et al., 2003, 2005; Mravec et al., 2009; Titapiwatanakun et al., 2009; Péret et al., 2012), were investigated whether or not they modulate early auxin-induced reporter gene expression at 30 min (**Figure 1**).

We wanted to quantify transcription as a response to auxin so that, we compared transcriptional abundance at *t* = 0 min in the wt to transcription at 30 min (or 10 min, see below) after auxin addition. In the wt the fold transcriptional abundance of a reporter gene at *t* = 0 in comparison to the internal standard gene was determined and set as one fold. The fold expression change at *t* = 30 min in the wt was calculated in comparison to the *t* = 0 min value (being set as 1). This fold-induction by auxin corresponds to one bar for a reporter gene in the diagram for the wt (**Figure 1**). In the mutants the measurements and the calculations were done in the same way. Hence, for all reporter genes a series of fold-values was generated at 30 min for the wt and for all mutants. Comparing these patterns in the wt and mutants reflects the auxin responsiveness of transcription of the reporter genes in the lines at *t* = 30 min (or at *t* = 10 min, see below). Differences between the wt and the mutants of auxin in responsiveness can be detected even if the base value at *t* = 0 min is not identical in the wt and the mutants. The relative transcription of the reporter genes after 7 days of development in the mutants without auxin treatment indeed is not identical to the one at *t* = 30 min or *t* = 10 min (**Supplementary Figures S1**–**S6**). Of the *PIN* genes tested only transcription of *PIN3* turned out to be auxin-sensitive and transcription of *PIN1, PIN2*, and *PIN5* were not. On the other end of the auxin-sensitivity scale other genes were consistently found (*IAA19, SAU9, SAUR15, SAUR25, and GH3.5*), especially, when the total of results were compared (see below). Hence, the overall pattern of all measurements for each mutant of all reporter genes can be taken as a pattern of the fast responsiveness to auxin of that mutant. The slow response is the pattern of transcription after 7 days development (*t* = 0 min).

In *aux1/lax3* plants four reporter genes were transcriptionally up-regulated compared to the wt (*IAA2, IAA19, SAUR15, and SAUR23*). The overall pattern of expression in *aux1/lax3* mutants was different from and differences were greater than those in the other transport mutants. In *pin5* and *abcb1* the transcription of *IAA19* and *SAUR9* was increased compared to the wt, whereas *SAUR15*, *GH3.5*, and *PIN3* were down-regulated in response to auxin compared to the wt. Both patterns were similar. Auxin-induced transcription in *abcb19* was overall similar to *pin5* and *abcb1* but *SAUR9* and *SAUR15* were transcribed at the wt level.

We noticed that in the mutants used here not only the rapid auxin-induced expression was different from the wt but also the expression pattern of these reporter genes prior to auxin challenge, i.e., at *t* = 0 min (**Supplementary Figure S1**). Expression at *t* = 0 is equivalent to long-term developmental patterns in transcription of the reporter genes. When, we compared the wt *versus* mutants without auxin treatment (*t* = 0 min) (**Supplementary Figure S1**), we found that the mis-expression of reporter genes at *t* = 0 min among *pin5*, *abcb1*, and *abcb19* was remarkably similar, encompassing the same five reporter genes (*IAA2, IAA3, IAA14, IAA19, and PIN3*) and in *aux1/lax3*, again, *IAA2, IAA3, IAA14, SAUR9*, and *PIN3* (but not *IAA19*). Several more mis-regulated reporter genes, being different in different mutants, were also found in each of the transporter mutants.

### Protein Phosphatases and Kinases as Potential Auxin Signaling Intermediates Modulate Expression of Reporter Genes

A number of protein kinases and phosphatases (*cpk3, d6pk’s, ibr5*, and *rcn1*) are known to be auxin signaling mutants. CPK3 phosphorylated and activated pPLA enzymatic activity and *CPK3-OX* is an over-expressing line for this gene (Mehlmer et al., 2010; Rietz et al., 2010). The weak auxin signaling mutant *ibr5* (Monroe-Augustus et al., 2003; Strader et al., 2008) and the gravitropic mutant *rcn1* are protein phosphatase mutants (Garbers et al., 1996). Further kinases having functions in the regulation of PIN polarity and activity are the D6P kinases (Zourelidou et al., 2009, 2014). Therefore, the respective *D6PK* mutants were investigated.

**FIGURE 2:**
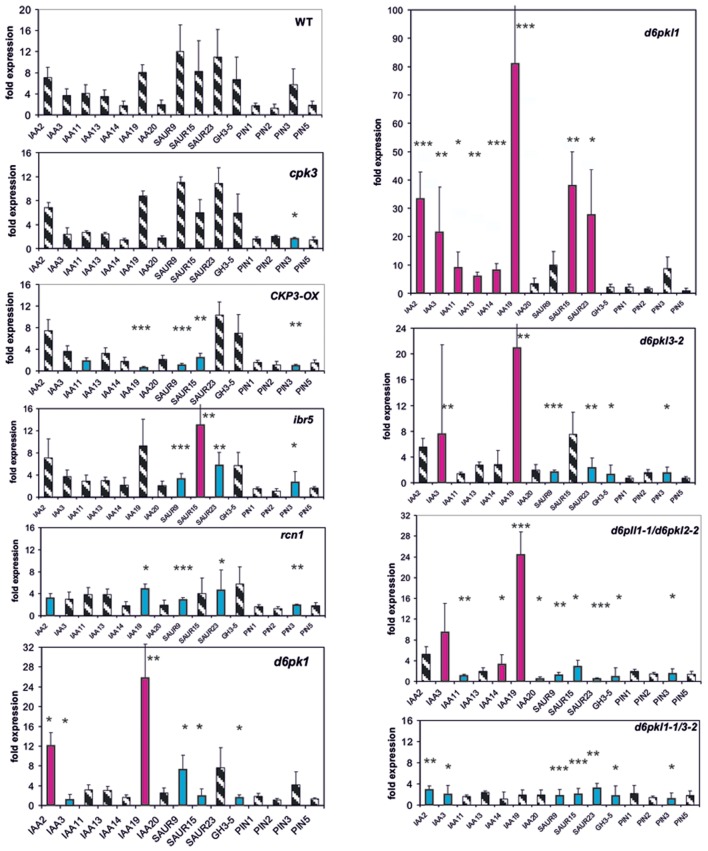
**Auxin-induced transcription of reporter genes in seedlings of mutants *cpk3*, the over-expressor *CPK3-OX, ibr5, rcn1, d6pk-1, d6pkl-1-1, d6pkl3-2, d6pkl-1/d6pkl3-2, d6pkl1-1/d6pk3-2*, and wt seedlings.** Seedlings were grown in the light for 7 days and auxin treatment was 30 min. For each, the wt and the mutants, one panel of the reporter genes is shown. Red bars indicate significant increased of transcription and blue bars significantly decreased transcription of reporter genes in a comparison between the wt and mutant. Striped bars indicate no significant difference between the wt and the mutant. Transcription in the untreated wt and mutants at *t* = 0 min were set as 1 and values after 10 μM treatment by IAA at *t* = 30 min were calculated relative to these values and represent as one bar either in the wt diagram or in a mutant diagram [^∗^*p* < 0.05, ^∗∗^*p* < 0.01, ^∗∗∗^*p* < 0.001; *t*-test].

Whereas in the *cpk3* mutant only one reporter gene, *PIN3*, was mis-expressed in *CPK3-OX*, in *ibr5* and *rcn1* four to five genes were mis-expressed after 30 min (**Figure 2**). In all, the differences as compared to the wt were small. The only gene which was mis-regulated in all four mutants was *SAUR23*. At *t* = 0, in *cpk3*, *CPK3-OX, ibr5*, and *rcn1* only 1–4 reporter genes were mis-regulated (**Supplementary Figure S2**).

**FIGURE 3:**
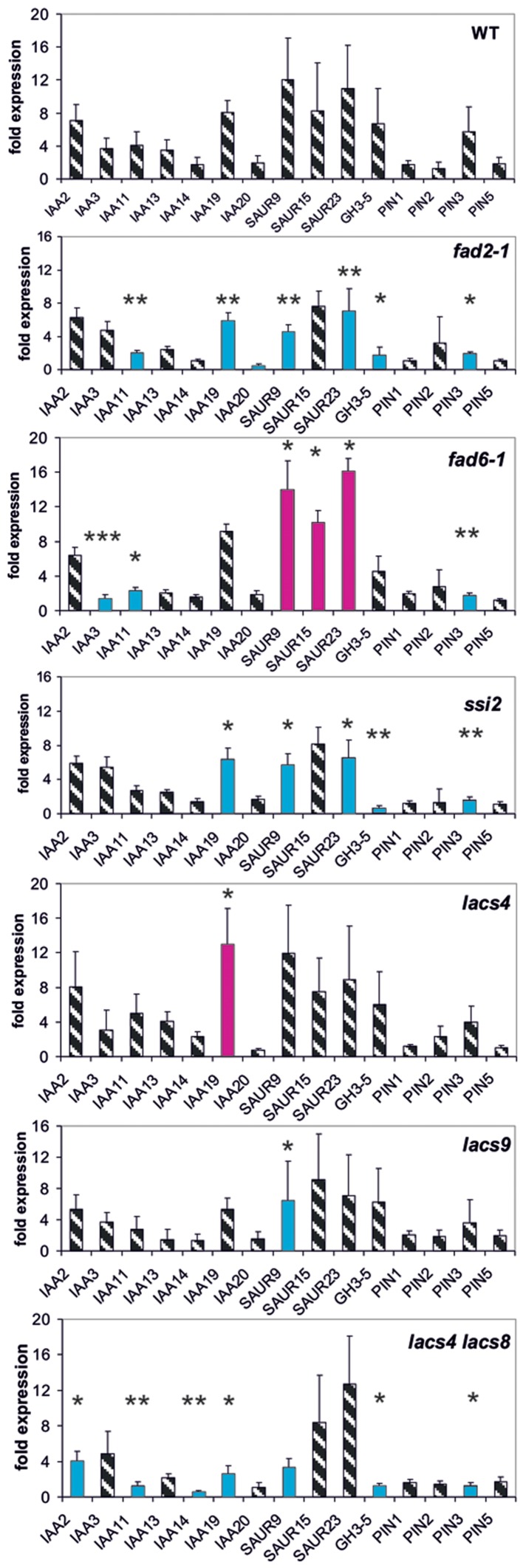
**Auxin-induced transcription of reporter genes in seedlings of the mutants *fad2-1, fad6-1, ssi2, lacs4, lacs9, lac4/lacs9*, and wt seedlings.** Seedlings were grown in the light for 7 days and auxin treatment was 30 min. For each of the wt and the mutants, one panel for the reporter genes is shown. Red bars indicate significant increased transcription and blue bars significantly decreased transcription of reporter genes in a comparison between the wt and mutant. Striped bars indicate no significant difference between the wt and the mutant. Transcription in the untreated wt and mutants at *t* = 0 min were set as 1 and values after 10 μM treatment with IAA at *t* = 30 min were calculated relative to these values and represent as one bar either in the wt diagram or in a mutant diagram [^∗^*p* < 0.05, ^∗∗^*p* < 0.01, ^∗∗∗^*p* < 0.001; *t*-test].

In the second group of protein kinase mutants (*d6pk-1, d6pkl-1, d6pkl3-2* and the double mutants *d6pkl1-1/2-2* and *d6pkl1-1/d6pkl3-2*) mis-expression of 6–10 reporter genes after 30 min was found to be substantially different from the wt (**Figure 3**). In contrast to most other mutants investigated, in the mutant *d6pkl-1* mis-expression of *IAA* genes was not delayed but always higher than in the wt after 30 min. At *t* = 0 min in the mutants of D6PK or D6PKL kinases the number of mis-expressed genes was 9–13 reporter genes (**Supplementary Figure S2**), which probably is related to the fact that the second group of protein kinases, the D6PKs, has different functions in auxin-signaling (Zourelidou et al., 2009, 2014).

### Lipid Metabolism Enzymes Interfere with Transcription of Reporter Genes

Another group of proteins linked to pPLA activation by auxin could be enzymes of lipid metabolism whose mutations lead to an altered membrane lipid composition compared to the wild type (wt; *fad2-1, fad6-1, ssi2, lacs4*, and *lacs9*) (Wallis and Browse, 2002; Kachroo et al., 2003; Zhang et al., 2009, 2012). Likewise, such mutations of lipid metabolism could highlight the importance of membrane lipid composition for the activity of transport proteins like PINs as a more indirect influence on auxin responsiveness (Roudier et al., 2010; Markham et al., 2011).

The single mutants *lacs4* and *lacs9* proved to be only weak mutants in which only one reporter gene was mis-regulated (**Figure 3**). In the mutants *fad2-1*, *ssi2* and in the *lacs4/lacs9* double mutant, we observed similarities in the patterns of mis-regulation in *IAA19, SAUR9, GH3.5*, and *PIN3* at 30 min (**Figure 3**). In *fad6-1* up-regulation of transcription was observed in the three *SAUR* genes.

Surprisingly, at *t* = 0 min in the single *lacs* mutants five genes and in the double mutant expression of 10 reporter genes was differentially regulated (**Supplementary Figure S3**), and four among them were identical in each mutant (*IAA14, IAA20, SAUR15*, and *SAUR23*). This may indicate again a similar functional impact of these long chain acyltransferases on certain auxin functions like auxin transport.

### Receptor Mutants of the TIR1/AFB Family Modulate Reporter Genes Weakly

**FIGURE 4:**
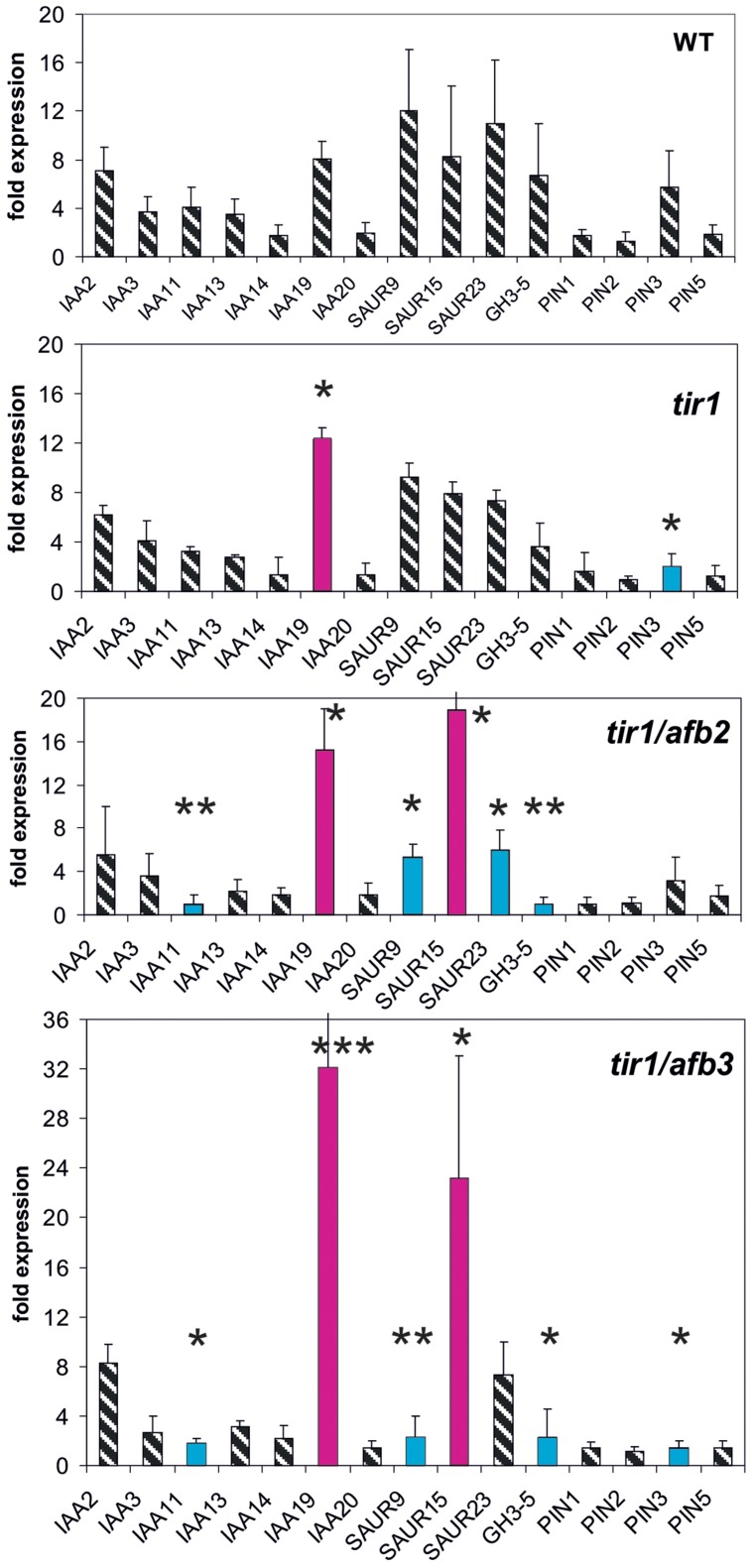
**Auxin-induced transcription of reporter genes in seedlings of the mutants *tir1, tir1/afb2, tir1/afb3*, and wt seedlings.** Seedlings were grown in the light for 7 days and auxin treatment was 30 min. For each the wt and of the mutants one panel for the reporter genes is shown. Red bars indicate significant increased transcription and blue bars significantly decreased transcription of reporter genes in a comparison between the wt and mutant. Striped bars indicate no significant difference between the wt and the mutant. Transcription in the untreated wt and mutants at *t* = 0 min were set as 1 and values after 10 μM treatment by IAA at *t* = 30 min were calculated relative to these values and represent as one bar either in the wt diagram or in a mutant diagram [^∗^*p* < 0.05, ^∗∗^*p* < 0.01, ^∗∗∗^*p* < 0.001; *t*-test].

Finally, we investigated the reporter gene expression in the auxin receptor mutants *tir1, tir1/afb2*, and *tir1/afb3* (**Figure 4**). The *TIR1/AFB* gene family encompasses six genes and their expression is not regulated by auxin (Parry et al., 2009). After 30 min auxin treatment only two genes were weakly mis-expressed in *tir1* (**Figure 4**). At *t* = 30 min mis-expression was almost identical in both double mutants *tir1/afb2* and *tir1/afb3* affecting 6–7 reporter genes (*IAA11, IAA19, SAUR9, SAUR13, SAUR15*, and *PIN3*). The magnitude of mis-expression was considerably higher in the double mutants as compared to the single mutant *tir1*, and transcription of two reporter genes were up-regulated, not down-regulated (*IAA19, SAUR25*). Mis-expressed reporter genes at *t* = 0 min were again dissimilar to those at *t* = 30 min (**Supplementary Figure S4**). No reporter gene was found to be mis-expressed in *tir1* at *t* = 0 min, and 5–6 reporter genes in the receptor double mutants *tir1afb2* and *tir1afb3*.

### In Mutants Early Auxin-Induced Gene Expression Is Modulated Already at 10 min

**FIGURE 5:**
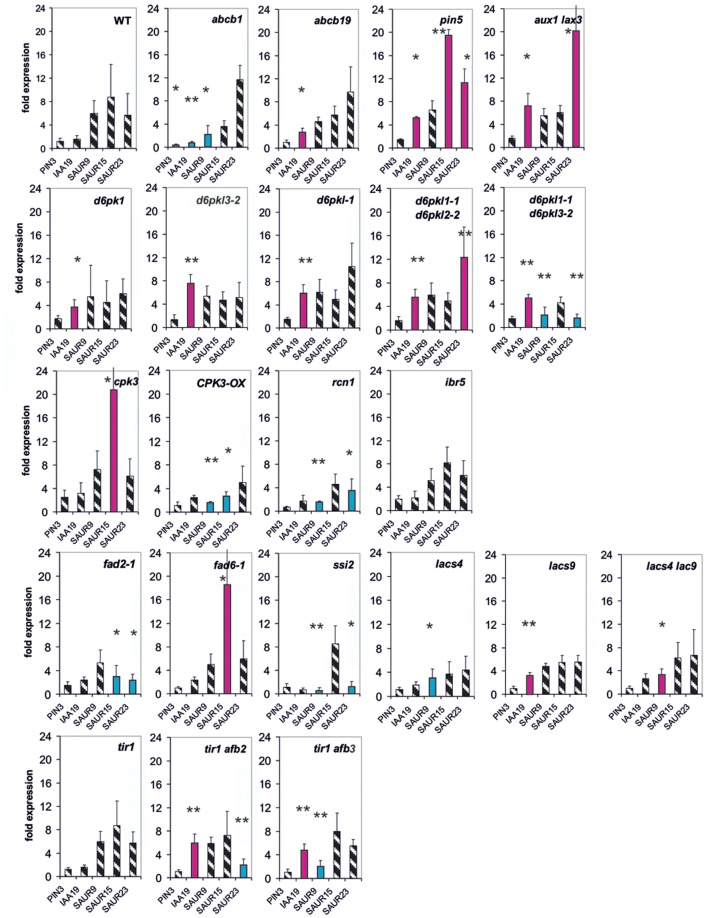
**Auxin-induced transcription of selected reporter genes in seedlings of all mutants used in this study and in wt seedlings.** Seedlings were grown in the light for 7 days and auxin treatment was for 10 min. For each wt and of the mutants a panel for the reporter gene is shown. Red bars indicate significant increased transcription and blue bars significantly decreased transcription of reporter genes in a comparison between the wt and mutant. Striped bars indicate no significant difference between the wt and the mutant. Transcription in the untreated wt and mutants at *t* = 0 min were set as 1 and values after 10 μM treatment by IAA at *t* = 10 min were calculated relative to these values and represent one bar either in the wt diagram or in a mutant diagram [^∗^*p* < 0.05, ^∗∗^*p* < 0.01, *t*-test].

All genes represented by the mutants investigated here were not transcriptionally regulated by auxin for at least 1 h post treatment (**Supplementary Figure S5**, Nemhauser et al., 2006). After 4 h auxin treatment of *Arabidopsis* root tips the RNAs were analysed by second generation sequencing and the HTSeq software (Anders et al., 2015) and all genes defined by the mutants were found not to be regulated by in their transcription (unpublished data from Paulo Teixeira and Alan M. Jones, University of North Carolina). Nevertheless, regulation of transcription of test genes could be due to fast transcription and translation of another protein, which then could regulate TIR1/AFB activities. Therefore, we chose to select only five test genes with very rapid regulation of transcription out of our sample and quantify their auxin induction after 10 min (**Figure 5**). Ten minutes seems to be an extremely short time for expression of any protein that would have a function on TIR1/AFBs in the nucleus strongly enough to affect an auxin-induced readout. Therefore this time point seems suitable to exclude a transcriptional/translational back-coupling mechanism when starting with auxin binding to TIR1/AFBs.

At *t* = 10 min, we measured expression of five genes (*PIN3, IAA19, SAUR9, SAUR15*, and *SAUR23*), chosen for their rapidity to respond to an auxin stimulus (**Figure 5**). In 20 out of 22 mutants at least one reporter gene was mis-regulated already after 10 min (**Figure 5**), and in most cases several were mis-regulated. In 14 mutants *IAA19* and/or *SAUR* genes were transcriptionally increased compared to the wt at 10 min (*lacs9, d6pk-1, tir1afb2*, and *tir1afb3*). In *abcb1* the induction of *IAA19* expression by auxin was decreased and in *abcb19* it was increased over the wt. Transcription of no reporter gene was significantly different from the wt at 10 min in the mutants *ibr5* and *tir1*. Hence, already at 10 min in the great majority of all mutants at least one reporter and often 2–3 reporter genes were mis-regulated at a significant level. As could be expected the extent of mis-regulation at 10 min was generally smaller than at 30 min. Another aspect is that at *t* = 10 min over-shooting of the reporter transcription (as compared to the wt) was found more often than at *t* = 30 min.

However, with the exception of *PIN3* after *t* = 30 min, the expression of none of the mutant genes was up-regulated by auxin even for at least 1 h post auxin treatment (**Supplementary Figure S5** and unpublished data from Paulo Teixeira and Alan M. Jones, University of North Carolina). This makes it clear that transcriptional regulation of the genes represented by the mutants was not the reason for the fast modulation of the TIR1/AFB-dependent readout. Expression of only some mutant genes was increased by auxin after 3 h (**Supplementary Figure S5**; *IBR5, D6PK-1*, and *D6PK-2*), which still excludes an effect of those mutant proteins/genes on TIR1-directed expression of reporter genes at 30 min. We emphasize that our data are demonstrating for the 10-min auxin response, a receptor is necessary (**Figure 5**).

### Comparison of Long-Term Expression of Reporter Genes to their Short Term Induction by Auxin

**FIGURE 6:**
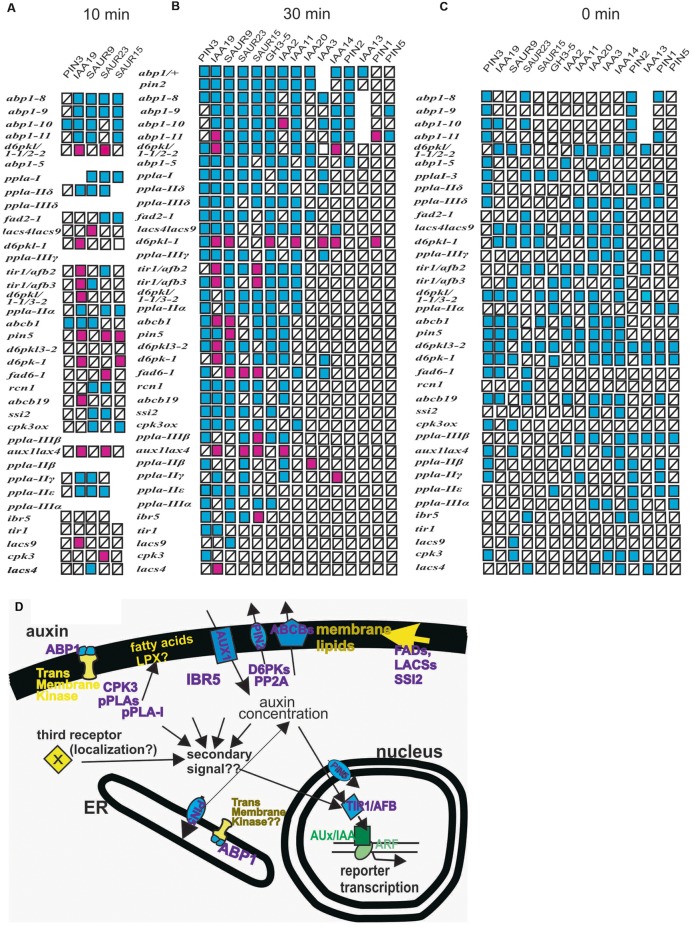
**(A–C)** Summary of auxin-induced transcription in mutants and the number of defects in early auxin-induced gene expression. Red squares represent increased transcription as compared to the wt and blue squares represent decreased values than those found in the wt. Squares with a black bar represent transcription not significantly different from the wt. Previously published data are incorporated into the figure (Effendi and Scherer, 2011; Effendi et al., 2011, 2013, 2014; Labusch et al., 2013). To allow for a synopsis and a comparative analysis, we assembled all previously published results on *ppla* and *abp1* mutants (Effendi et al., 2013, 2014, 2015; Labusch et al., 2013) and the experiments here into one scheme. The mis-expression of the reporter genes in plants after 10 min of auxin treatment (**Figure 6A**), after 30 min of 10 μM auxin treatment (**Figure 6B**), and at *t* = 0 min are presented (**Figure 6C**). Data on *ppla* mutants at *t* = 0 (**Supplementary Figure S6**) are included into **Figure 6C**. Mutants were sorted vertically by the number of mis-regulated reporter genes. Reporter genes were sorted vertically according to the number of deviations from the wt. Values that were not quantified are left as empty spaces. In the schemes for *t* = 0 min and *t* = 10 min, this arrangement is done accordingly. Only *tir1, lacs4, lacs8*, and *cpk3* were weak mutants in this auxin-induced transcription test. **(D)** The mutant gene products are arranged into a schematic cell to indicate their subcellular localization. A hypothetical linkage between TIR1 and ABP1 is indicated by arrows. Also, the possibility of a hypothetical third receptor with unknown localization is indicated.

To allow a synopsis and a comparative analysis, we assembled all previously published results on *ppla* and *abp1* mutants (Effendi et al., 2013, 2014, 2015; Labusch et al., 2013) and the experiments here into one simplifying scheme (**Figure 6**) of the mis-expression of the reporter genes after 10 (**Figure 6A**) and 30 min of 10 μM auxin treatment (**Figure 6B**) and at *t* = 0 min (**Figure 6C**). The subcellular localization of the mutant gene products is summarized in **Figure 6D**. Previously unpublished data on *ppla* mutants at *t* = 0 (**Supplementary Figure S6**) are incorporated into **Figure 6C**. The mutant lines are arranged vertically according to the number of mis-regulated reporter genes after 30 min of auxin application (**Figure 6B**), and the list of reporter genes is arranged with the most frequently mis-regulated reporter genes on the left side. In the scheme for *t* = 10 min (**Figure 6A**) and *t* = 0 min (**Figure 6C**) the genes were arranged accordingly. In the pplaI mutant, we had found eight mis-regulated reporter genes (Labusch et al., 2013; Effendi et al., 2014) and in the other *ppla* mutants four to seven at *t* = 30 min (Labusch et al., 2013; Effendi et al., 2014). All *abp1* mutants had high scores of mis-regulated reporter genes at *t* = 30 min (8 in *abp1–5* and 9–12 in the engineered *abp1* mutants at *t* = 30 min). At *t* = 10 the mutants *abp1–8, abp1–9, abp1–10*, and *abp1–11* transcribed the reporter genes generally at a lower rate (**Figure 6B**; and data from Effendi et al., 2015). As stated before, the only weak mutants in our auxin-induced transcription test (*t* = 30 min) were *tir1, lacs4, lacs8*, and *cpk3*, probably because all of them are members of gene families so that genetic redundancy could play a role to reduce the impact of a single gene mutant.

Another aspect is that over-shooting of transcription of reporter genes in response to auxin is more frequent at *t* = 10 min (18 out of 34 responding reporter genes), than at *t* = 30 min (22 of 122 responding reporter genes). No over-shooting of reporter genes was found at *t* = 0 min, which, however, is the consequence of 7 days development with a mutant genome. This indicates a time dependence, which, in view of the absence of a mechanistic model for TIR1 activity regulation, remains unexplained. It may rely again on the differing contributions of different members of the TIR1/AFB gene family due to genetic redundancy.

## Discussion

### Transcriptional Readout Controlled by the TIR1/AFB Receptors Is an Auxin Response

Transcription of early auxin-induced genes is a valid auxin response. The reporter genes, we chose clearly displayed a regulatory pattern. Some genes such as *PIN1, PIN2*, and *PIN5* are non-responsive, others like *IAA19, SAUR9, SAUR15*, and *SAUR25* are highly and rapidly responsive (**Figures 5** and **6A,B**) so that, as a whole, a clear pattern of fast responsiveness in the wt and in the mutants becomes apparent. This pattern of responsiveness is not identical to the pattern generated from transcription of the reporter genes at *t* = 0, which corresponds to the changes in gene expression during 7 days seedling development (**Figure 6C**). It should be noted that already the mis-regulation of only one reporter gene in only one mutant would mean that, we would have to conclude that an auxin receptor-driven process depending on this mutant gene product causes this. In fact, we found several mis-regulated genes in many mutants. Hence, deviations in single values do not undermine this hypothesis, the complete pattern is the true argument for it. The measurements at *t* = 30 min argue for a receptor-driven pathway to regulate TIR1/AFB activities with special emphasis for all mutant gene products which are membrane proteins (**Figure 6D**). This is given by the fact that membrane protein amounts are not rapidly changed by transcription/translation. Their expression at the plasma membrane needs 1 h or longer (Scherer, 2011). Changes in transcription of *ABP1, PIN2, PIN3, ABCB1, ABCB19*, and *AUX1/LAX3* cannot affect expression at the plasma membrane within 30 min to cause back-coupling to TIR1. Similarly, it seems very unlikely that changes in membrane lipid composition will be measurable after 30 min transcription of genes of reporter genes in *LACS4, LACS9, SSI2, FAD2-1*, or *FAD6-1* mutants. Lipid compositions are the target of these genes so that their changes could result in activity changes of membrane proteins, e.g., transport proteins which are influenced by lipid composition so that timing, either 10 min or 30 min auxin treatment, is not the point (Roudier et al., 2010; Markham et al., 2011).

Almost all mutants employed in this and our previous studies concern genes known to have functions in auxin signaling (see references in **Supplementary Table S1**). Only few have not yet been investigated in detail as potential auxin signaling mutants like *cpk3, CPK3-OX*, and some *pplas* but actually only *cpk3* was truly similar to the wt in its transcriptional responsiveness at the 10 and 30 min (**Figures 2** and **5**). Another such example is *tir1*, which is also very similar to the wt in our results, probably because of the genetic redundancy of this receptor family (**Figure 4**).

Transcription of all of the genes defined by the mutants is not modulated by auxin for at least 1 h with the interesting exception of ABP1 (Effendi et al., 2015). To increase of the amount of ABP1 in the plasma membrane, however, takes considerably longer than the first effects on transcription of *ABP1* because the time for transport to the plasma membrane is at least 1 h (Scherer, 2011). Therefore, transcriptional feedback or feedforward through these mutant genes is not possible in less than an hour. Transcription/translation, however, is the only mechanism for signal propagation available to the TIR1/AFB receptors (Mockaitis and Estelle, 2008). We found aberrant transcription of 2–3 reporter genes already in 11 mutant lines after 10 min and of one reporter genes in nine mutant lines out of 22 total (**Figure 5**). Therefore, we must conclude that the output of TIR1/AFBs is modulated within 10 min by an auxin-initiated, receptor-driven process.

Can TIR1/AFBs be regulated within 10 min? In our previous report measuring the degradation of an Aux/IAA1-luciferase hybrid protein, we showed with this method modulation of TIR1 activity after 4 min by inhibitors of pPLA (Scherer et al., 2007). Yu et al. (2015) showed that enhancing the untethering of TIR1 from the SCF complex inhibits TIR1 activity so that this reaction could provide a model idea to regulate TIR1 enzymatic activity. In a speculative review, neddylation of the SCF/TIR1 complex was hypothesized to be a mechanism of regulation of TIR1 so that potential regulation of TIR1 receptors cannot be excluded (Parry and Estelle, 2004).

The identity of the transcriptional/translational mechanism, starting with TIR1/AFB auxin perception, that can so rapidly regulate TIR1 activity remains unknown. It would require an auxin-induced, rapid biosynthesis of a protein (i.e., a translational mechanism) that is capable of changing TIR1/AFB activities. But such a protein has yet to be found.

Logically, the auxin-induced transcriptional response of reporter genes requires a receptor to initiate such fast downstream responses (Scherer et al., 2012) even if regulation would affect solely mRNA levels. This could be an as yet unknown auxin receptor or even ABP1. ABP1 binds to four transmembrane kinases, which can activate RIC/ROP signaling (Dai et al., 2013; Xu et al., 2014) and could employ a post-translational mechanism, which can operate on the time scale of minutes. Conceivably, a receptor kinase could phosphorylate various proteins or initiate phosphorylation cascades so that signal transmission within minutes into the cytosol would be a possibility. It should also be kept in mind that numerous auxin responses are known, which have been detected in fewer than 10 min (Scherer, 2011) and which are difficult to reconcile with statements that ABP1 has no function in auxin signaling (Gao et al., 2015). This can be said despite the recent revelation that defects in the neighboring *BSM* gene cause the embryo-lethal phenotype in *abp1–1* (Dai et al., 2015; Michalko et al., 2015). Other rapid responses to auxin are still linked to a functional ABP1 (Robert et al., 2010; Xu et al., 2010, 2014; Dai et al., 2013). This statement is valid in spite of the equally surprising failure of the ethanol-inducible repression of ABP1 function by an antibody fragment (Michalko et al., 2016). We conclude that, we can neither completely disprove TIR1/AFBs to be the receptors for the observed changes in auxin-induced responsiveness of transcription nor can we completely prove that ABP1 is the receptor for that response. The possibility that a third receptor has this function, however, seems remote.

## Author Contributions

CL designed research and experiments. YE designed research and experiments. MF provided material and wrote article. GS designed Research and wrote article.

## Conflict of Interest Statement

The authors declare that the research was conducted in the absence of any commercial or financial relationships that could be construed as a potential conflict of interest.

## Footnotes

1 http://jsp.weigelworld.org/expviz/expviz.jsp

## Abbreviations

ABP1: AUXIN BINDING PROTEIN1
AFBs: AUXIN SIGNALING F-BOX proteins
Aux/IAA protein: Auxin/indoleacetic acid protein
PIN protein: PIN-FORMED protein
pPLA: patatin-related Phospholipase A
SAUR: small auxin-up RNA
TIR1: TRANSPORT INHIBITOR RESPONSE 1
