# Supplementary material for: Transcription of TIR1-Controlled Genes Can be Regulated within 10 Min by an Auxin-Induced Process. Can TIR1 be the Receptor?
Source: Front Plant Sci. 2016 Jul 11;7:995. doi: 10.3389/fpls.2016.00995 (PMC4939301; doi:10.3389/fpls.2016.00995)
Supplement: Supplementary file 1 [file Data_Sheet_1.PDF]

Supplemental Figures

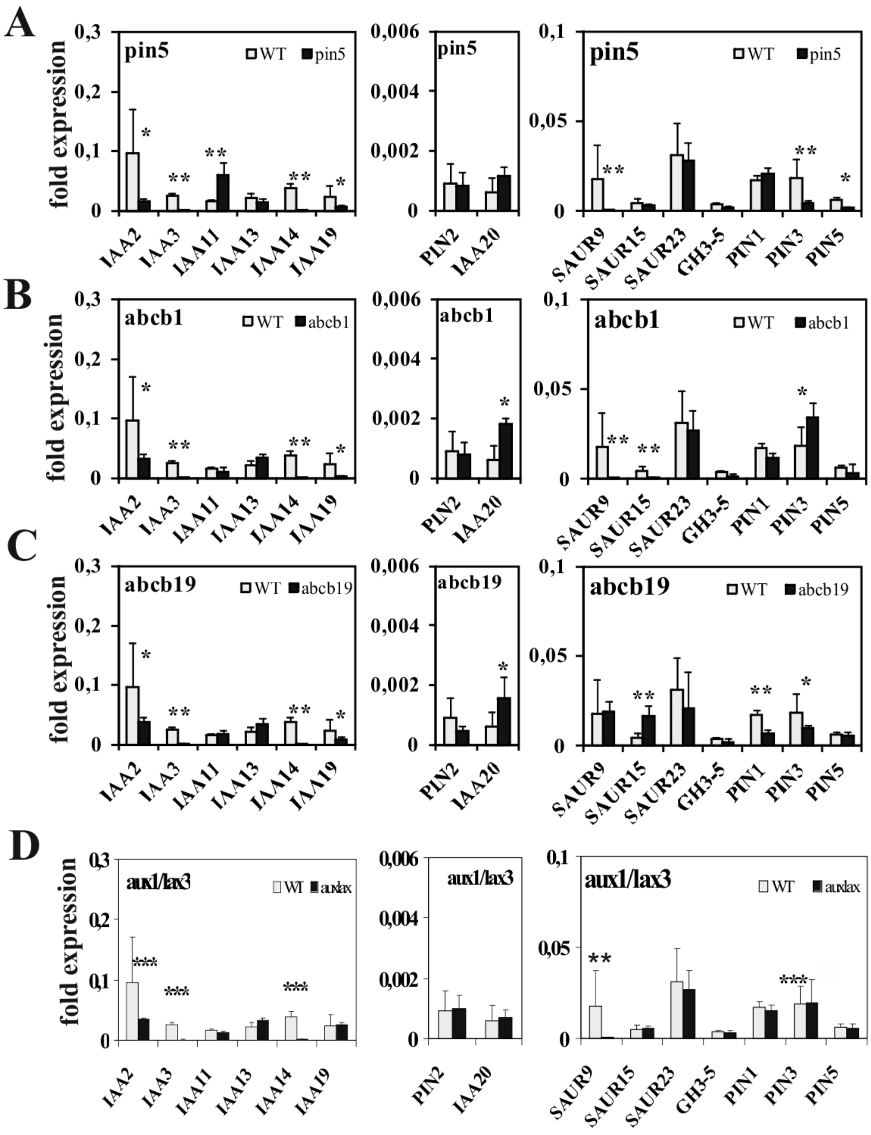

Supplemental Figure S1. Expression of auxin transporter genes without auxin treatment. Expression of early auxin-induced marker genes in the light-grown seedlings of mutants *pin5, abcb1, abcb19, aux1/lax3* and wild type seedlings prior to auxin treatment. Expression of untreated wt and mutants at t=0 min were calculated relative to the same reference gene *UBQ10.* White bars: wild type; black bars: mutant as indicated. ((*): p < 0.05, (**): p < 0.01, (***): p < 0,001; t-test).

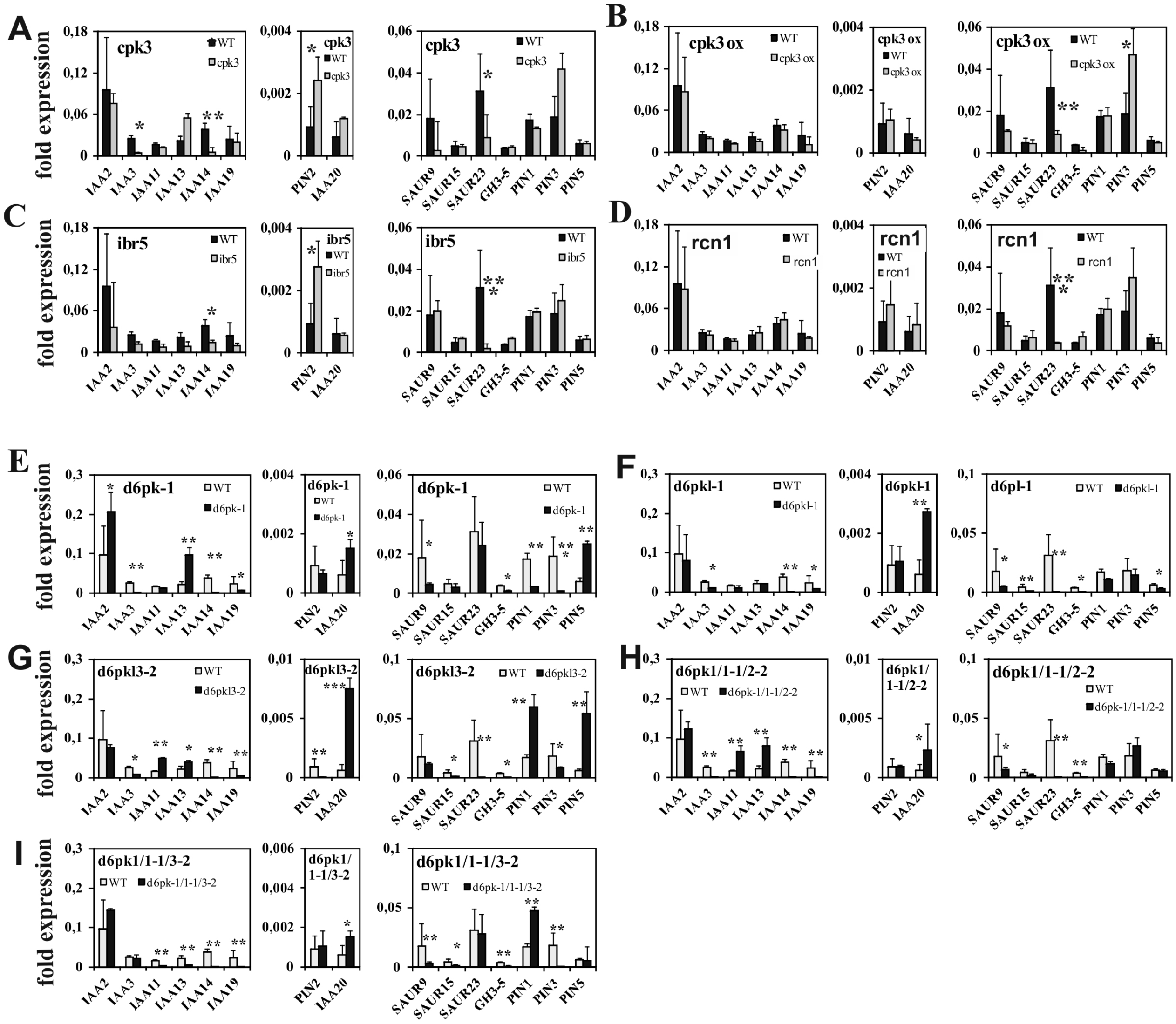

Supplemental Figure S2. Expression of protein phosphatase and kinase genes without auxin treatment. Expression of early auxin-induced marker genes in the light-grown seedlings of (A-D) *cpk3*, over-expressor *CPK3-OX*, *ibr5* and *rcn1* and wild type seedlings and in (E-I) *d6pk-1, d6pkl-1-1, d6pkl3-2, d6pkl-1/d6pkl3-2, d6pkl1-1/d6pk3-2* and wild type seedlings, all prior to auxin treatment. Expression of untreated wt and mutants at t=0 min were calculated relative to the same reference gene *UB10.* Wild type (white bars) and mutants (black bars) are indicated in the panels. Expression of untreated wt and mutants at t=0 min were calculated relative to the same reference gene *UBQ10*. ((*): p < 0.05, (**): p < 0.01, (***): p < 0,001; t-test).

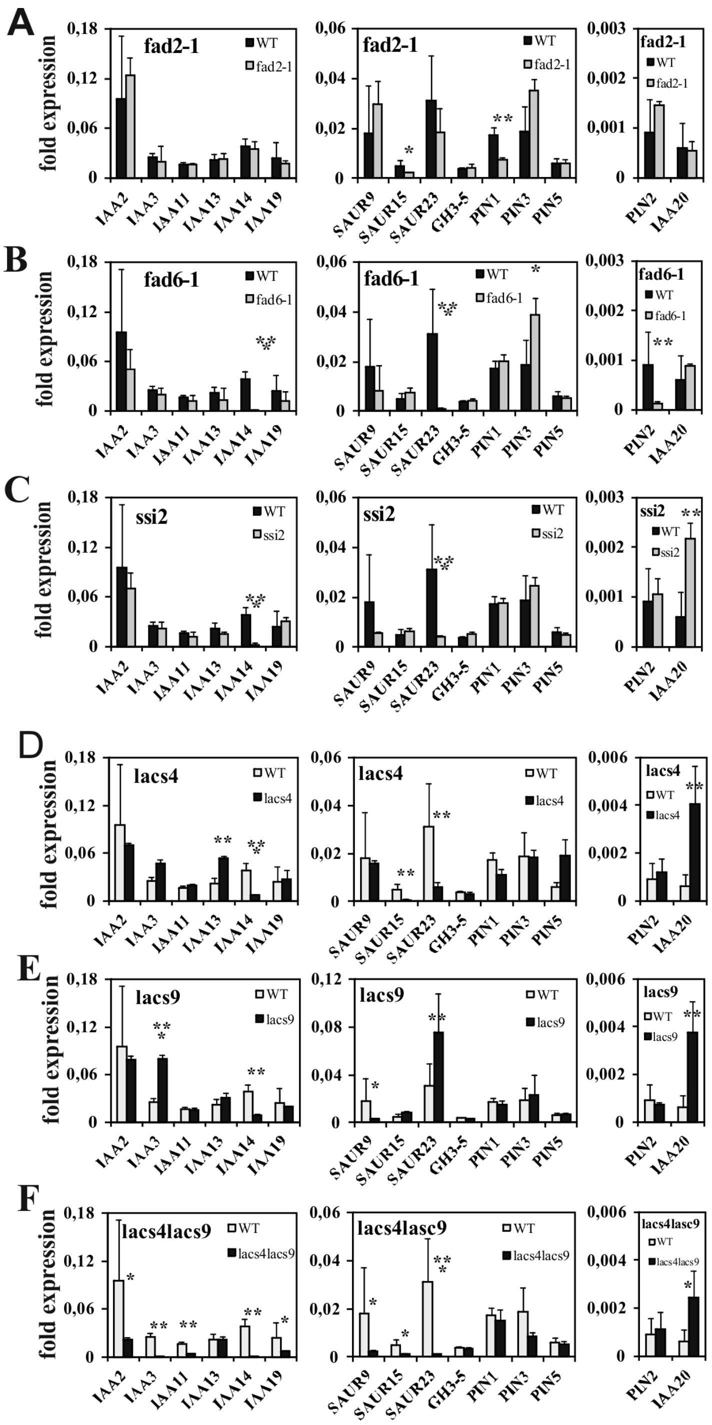

Supplemental Figure S3. Expression of genes in lipid metabolism without auxin treatment. Expression of early auxin-induced marker genes in the light-grown seedlings of mutants (A) *fad2-1*, *fad6-1* and *ssi2* and mutants (B) *lacs4, lacs9, lac4/lacs9* and wild type seedlings, all prior to auxin treatment. Expression of untreated wt and mutants at t=0 min were calculated relative to the same reference gene *UB10.* Mutants are indicated in the panels. Expression of untreated wt and mutants at t=0 min were calculated relative to the same reference gene *UBQ10*. ((*): $p < 0.05$, (**): $p < 0.01$, (***): $p < 0,001$; t-test).

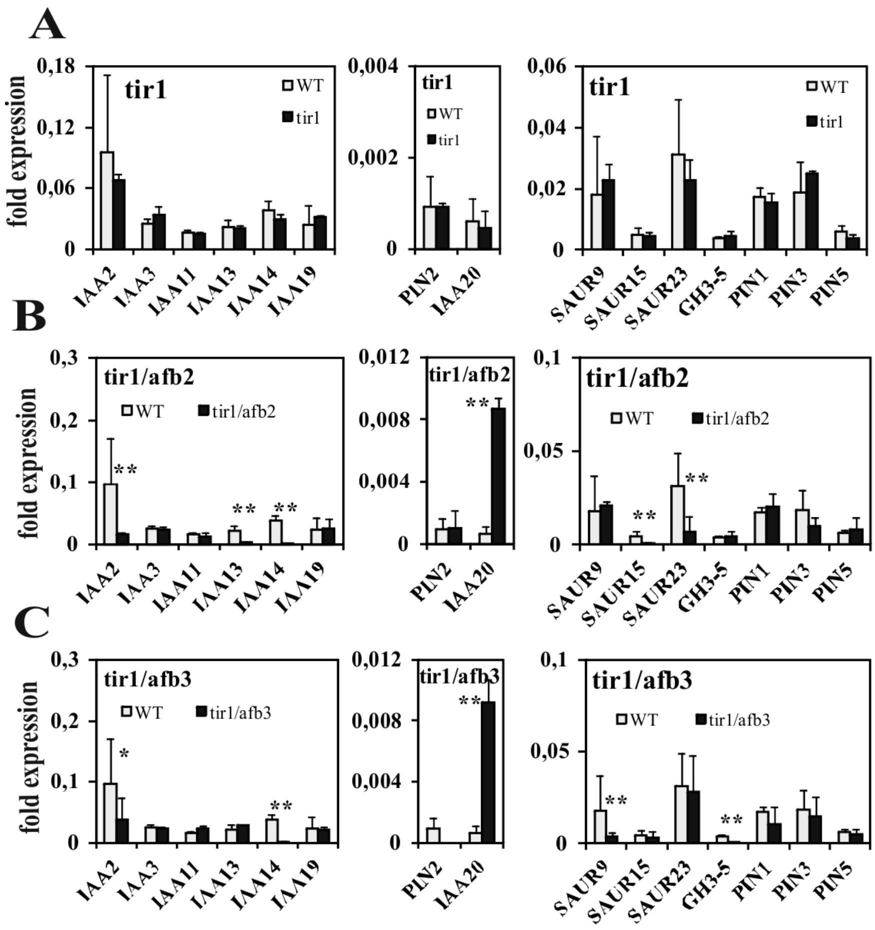

Supplemental Figure S4. Expression of TIR1-related genes without auxin treatment. Expression of early auxin-induced marker genes in the light-grown seedlings of mutants *tir1*, *tir1/afb2*, *tir1/afb3* and wild type seedlings, all prior to auxin treatment. Expression of untreated wt and mutants at t=0 min were calculated relative to the same reference gene *UB10.* Mutants are indicated in the panels. Expression of untreated wt and mutants at t=0 min were calculated relative to the same reference gene *UBQ10*. ((*): p < 0.05, (**): p < 0.01, (***): p < 0,001; t-test).

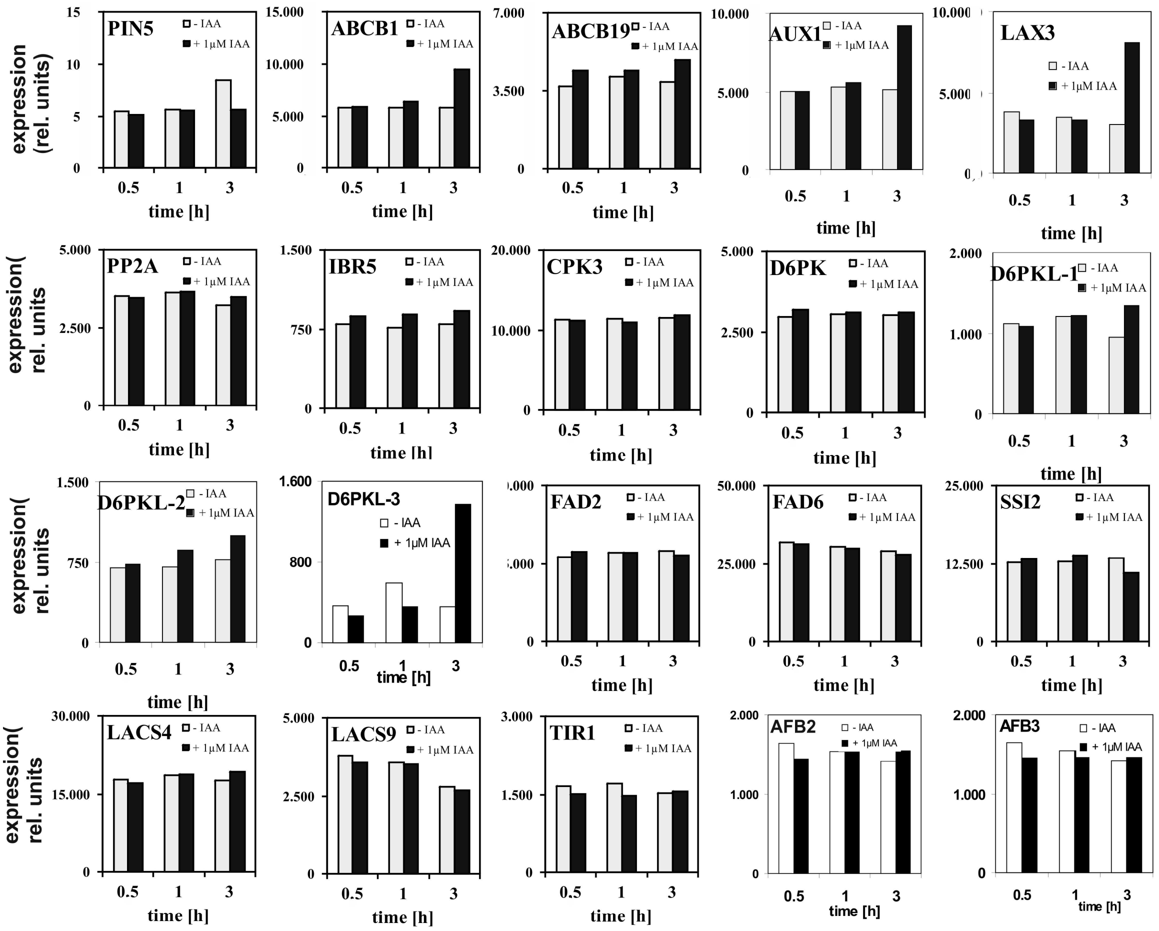

Supplemental Figure S5. Lack of auxin activation of the genes represented by the mutants. Time courses after auxin application of expression of those genes used as mutants in this study. Data are from AtGenExpress Visualisation Tool (controls: white bars; 1 µM auxin: black bars). (http://jsp.weigelworld.org/expviz/expviz.jsp) Nemhauser, J. L., Hong, F., and Chory, J. (2006). Different plant hormones regulate similar processes through largely nonoverlapping transcriptional responses. Cell 126, 467–475.)

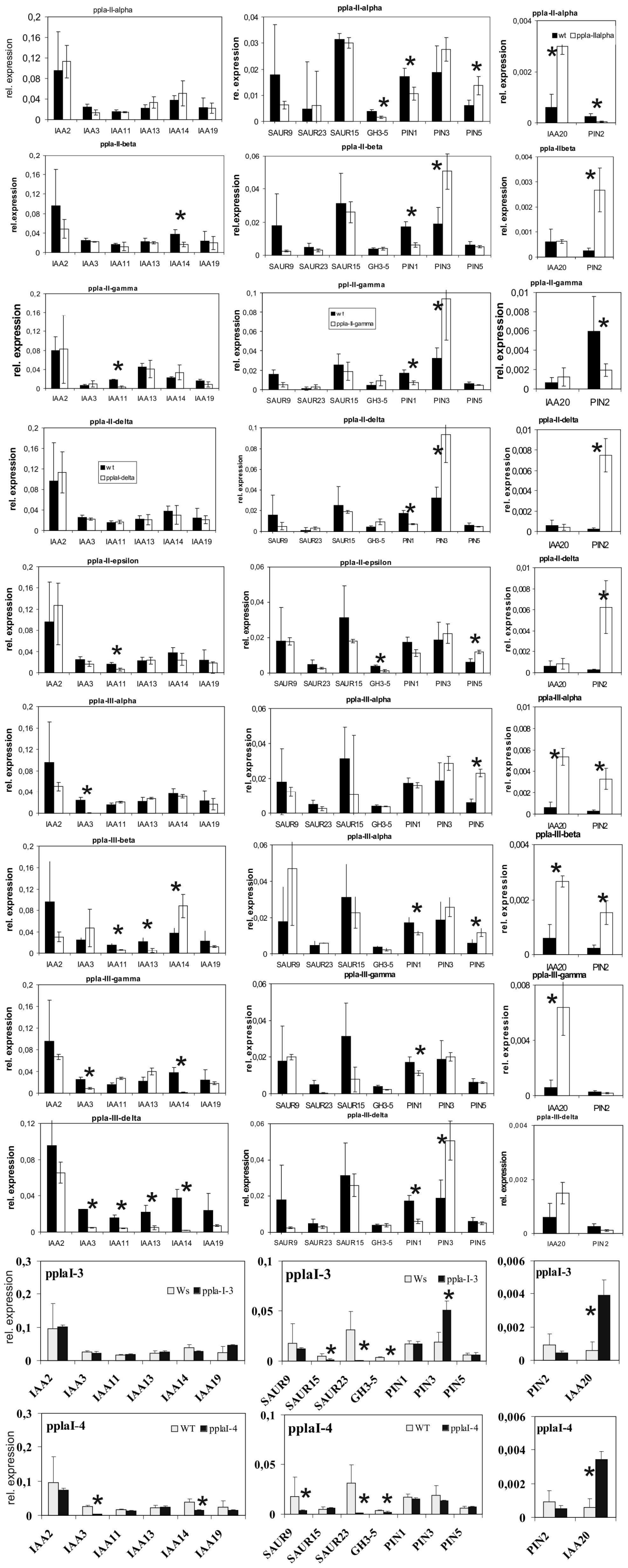

Supplemental Figure S6. Expression of auxin-induced marker genes in *ppla* mutants without auxin treatment. Transcriptional pattern at t=0 of previously published *ppla* mutants (Labusch et al. 2013). Mutants are indicated in the panels. Data are integrated into Fig. 6. Expression of untreated wt and mutants at t=0 min were calculated relative to the same reference gene *UB10*. ((*): $p < 0.05$, t-test).

Supplemental Table 1. Sources of mutant lines and references.

***pin5-5* (AT5G16530) were given to us by M. Geisler (Mravec et al. 2005).**

Mravec, J., Skůpa, P., Bailly, A., Hoyerová, K., Krecek, P., Bielach, A., Petrásek, J., Zhang, J., Gaykova, V., Stierhof, Y.D., Dobrev, P.I., Schwarzerová, K., Rolcík, J., Seifertová, D., Luschnig, C., Benková, E., Zazímalová, E., Geisler, M., and Friml, J. (2009). Subcellular homeostasis of phytohormone auxin is mediated by the ER-localized PIN5 transporter. Nature. 459, 1136-1140.

***abcb1-1*, (At3g28860) *abcb19-1* (At2g36910)**

Knockdown mutants *abcb1-1* and *abcb19-1*) and were given to us by M. Geisler (Noh et al. 2001, Geisler et al. 2003,Geisler et al. 2005):

Noh, B., Murphy, A.S. and Spalding, E.P. (2001). Multidrug resistance-like genes of Arabidopsis required for auxin transport and auxin-mediated development. Plant Cell. **13,** 2441–2454.

Geisler, M., Kolukisaoglu, H.U., Bouchard, R. Billion K, Berger J, Saal B, Frangne N, Koncz-Kalman Z, Koncz C, Dudler R, Blakeslee JJ, Murphy AS, Martinoia E, Schulz B. (2003). TWISTED DWARF1, a unique plasma membrane-anchored immunophilin-like protein, interacts with Arabidopsis multidrug resistance-like transporters AtPGP1 and At-PGP19. Mol. Biol. Cell. 14, 4238–4249.

Geisler, M., Blakeslee, J.J., Bouchard, R., Lee, O.R., Vincenzetti, V., Bandyopadhyay, A., Titapiwatanakun, B., Peer, W.A., Bailly, A., Richards, E.L., Ejendal, K.F., Smith, A.P., Baroux, C., Grossniklaus, U., Müller, A., Hrycyna, C.A., Dudler, R., Murphy, A.S., and Martinoia, E. (2005). Cellular efflux of auxin catalyzed by the Arabidopsis MDR/PGP transporter AtPGP1. Plant J. 44, 179-194.

***aux1-21/lax3* were given to us by R. Swarup** (Swarup et al. 2001; Péret et al. 2012).

aux1-21 (AT2G38120) *lax3* (AT1G77690)

Roman G, Lubarsky B, Kieber JJ, Rothenberg M, Ecker JR**.** (1995).Genetic analysis of ethylene signal transduction in *Arabidopsis thaliana*: five novel mutant loci integrated into a stress response pathway. Genetics. **139,** 1393–1409.

Swarup, R., Friml, J., Marchant, A., Ljung, K., Sandberg, G., Palme, K., and Bennett, M. (2001) Localization of the auxin permease AUX1 suggests two functionally distinct hormone transport pathways operate in the Arabidopsis root apex. Genes Dev. 15, 2648-2653.

Péret, B., Swarup, K., Ferguson, A., Seth, M., Yang, Y., Dhondt, S., James, N., Casimiro, I., Perry, P., Syed, A., Yang, H., Reemmer, J., Venison, E., Howells, C., Perez-Amador, M.A., Yun, J., Alonso, J., Beemster, G.T., Laplaze, L., Murphy, A., Bennett, M.J., Nielsen, E., and Swarup, R. (2012). AUX/LAX genes encode a family of auxin influx transporters that perform distinct functions during Arabidopsis development. Plant Cell. 24, 2874-2885.

Swarup K, Benková E, Swarup R, Casimiro I, Péret B, Yang Y, Parry G, Nielsen E, De Smet I, Vanneste S, Levesque MP, Carrier D, James N, Calvo V, Ljung K, Kramer E, Roberts R, Graham N, Marillonnet S, Patel K, Jones JD, Taylor CG, Schachtman DP, May S, Sandberg G, Benfey P, Friml J, Kerr I, Beeckman T, Laplaze L, Bennett MJ (2008). The auxin influx carrier LAX3 promotes lateral root emergence. Nat. Cell Biol**.** **10,** 946-954.

Péret, B., Swarup, K., Ferguson, A., Seth, M., Yang, Y., Dhondt, S., James, N., Casimiro, I., Perry, P., Syed, A., Yang, H., Reemmer, J., Venison, E., Howells, C., Perez-Amador, M.A., Yun, J., Alonso, J., Beemster, G.T., Laplaze, L., Murphy, A., Bennett, M.J., Nielsen, E., and Swarup, R**.** (2012). AUX/LAX genes encode a family of auxin influx transporters that perform distinct functions during Arabidopsis development. Plant Cell. 24, 2874-2885.

The *cpk3-2* T-DNA insertion line (SALK_022862) was obtained from the SALK collection (Columbia ecotype). The ***CPK3* over-expresser** was obtained from Steffen Rietz from the University of Kiel.

Mehlmer N., Wurzinger B., Stael S., Hofmann-Rodrigues D., Csaszar E., Pfister B., Bayer R., Teige M. (2010). The Ca^{2+}-dependent protein kinase CPK3 is required for MAPK-independent salt-stress acclimation in Arabidopsis. Plant J, 63, 484–498.

The T-DNA insertion mutant *rcn1* (AT1G25490) represents a subunit of the protein phosphatase PP2A (Garbers et al. 1996) and was given to us by A. Delong.

Garbers, C., DeLong, A., Deruére, J., Bernasconi, P., and Söll, D. (1996). A mutation in protein phosphatase 2A regulatory subunit A affects auxin transport in Arabidopsis. EMBO J. 15, 2115-2124.

***ibr5-1* (AT2G04550)** (Monroe-Augustus et al. 2003) originated by mutagenesis.

Monroe-Augustus, M., Zolman, B.K., and Bartel, B. **(**2003). IBR5, a dual-specificity phosphatase-like protein modulating auxin and abscisic acid responsiveness in Arabidopsis. Plant Cell. 15, 2979-2991.

The d6pk lines:

Zourelidou, M., Müller, I., Willige, B.C., Nill, C., Jikumaru, Y., Li, H., and Schwechheimer, C. (2009). The polarly localized D6 PROTEIN KINASE is required for efficient auxin transport in *Arabidopsis thaliana*. Development. 136, 627-636.

The mutants *fad2-1,* AT3G12120*, fad6-1*AT4G30950, *ssi2* AT2G43710 (Kachroo et al. 2003) are from mutagenesis screens:

Shockey, J.M., Fulda, M.S., and Browse, J.A. (2002). Arabidopsis contains nine long-chain acyl-coenzyme A synthetase genes that participate in fatty acid and glycerolipid metabolism. Plant Physiol. **129,** 1710–1722.

Wallis, J.G., and Browse, J. (2002). Mutants of Arabidopsis reveal many roles for membrane lipids. Prog. Lipid Res. 41, 254-278.

Kachroo, A., Lapchyk, L., Fukushige, H., Hildebrand, D., Klessig, D., and Kachroo, P. (2003). Plastidial fatty acid signaling modulates salicylic acid- and jasmonic acid-mediated defense pathways in the Arabidopsis ssi2 mutant. Plant Cell. 15, 2952-2965.

lacs4 **(At4g23850) (SALK_101543)** ***lacs9*** **(At1g77590) (SALK_111835):**

Jessen, D., Olbrich, A., Knufer, J., Kruger, A., Hoppert, M., Polle, A., and Fulda, M**.** (2011). Combined activity of LACS1 and LACS4 is required for proper pollen coat formation in Arabidopsis. Plant J**. 68,** 715-726.

Zhao, L., Katavic, V., Li, F., Haughn, G.W., and Kunst, L. (2010). Insertional mutant analysis reveals that long-chain acyl-CoA synthetase 1 (LACS1), but not LACS8, functionally overlaps with LACS9 in *Arabidopsis* seed oil biosynthesis. Plant J. 64, 1048–1058.

The mutants *tir1–10* (AT3G62980) and double mutants ***tir1-10/afb2–3*** (AT3G26810) and ***tir1-10/afb3–4*** **(**AT1G12820) carry T-DNA insertion mutants (Parry et al. 2009) and were given to us by M. Quint:

Parry, G., Calderon-Villalobos, L.I., Prigge, M., Peret, B., Dharmasiri, S., Itoh, H., Lechner, E., Gray, W.M., Bennett, M., and Estelle, M. (2009). Complex regulation of the TIR1/AFB family of auxin receptors. Proc. Natl. Acad. Sci. USA. 106, 22540-22545.

Supplemental Table 2. Primer list

Oligo name	Gene	Sequence (5`- 3`)
UBQ10_for	AT4G05320	GGCCTTGTATAATCCCTGATGAATAAG
UBQ10_rev	AT4G05320	AAAGAGATAACAGGAACGGAAACATAGT
IAA2_for	AT3G23030	GGTTGGCCACCAGTGAGATC
IAA2_rev	AT3G23030	AGCTCCGTCCATACTCACTTTCA
IAA3_for	AT1G04240	AACTGAAACATCCCCTCCTC
IAA3_rev	AT1G04240	CCATCTCTCTCAAAGTACTCTCC
IAA11_for	AT4G28640	CCTCCCTTCCCTCACAATCA
IAA11_rev	AT4G28640	AACCGCCTTCCATTTTCGA
IAA13_for	AT2G33310	CACGAAATCAAGAACCAAACGA
IAA13_rev	AT2G33310	CACCGTAACGTCGAAAAGAGATC
IAA14_for	AT4G14550	CCTTCTAAGCCTCCTGCTAAAGCAC
IAA14_rev	AT4G14550	CCATCCATGGAAACCTTCAC
IAA19_for	AT3G15540	GGTGACAACTGCGAATACGTTACC
IAA19_rev	AT3G15540	CCCGGTAGCATCCGATCTTTTCA
IAA20_for	AT2G46990	CAATATTTCAACGGTGGCTATGG
IAA20_rev	AT2G46990	GCCACATATTCCGCATCCTCT
SAUR9_for	AT4G36110	GACGTGCCAAAAGGTCACTT
SAUR9_rev	AT4G36110	AGTGAGACCCATCTCGTGCT
SAUR15_for	AT4G38850	ATGGCTTTTTTGAGGAGTTTCTTGGG
SAUR15_rev	AT4G38850	TCATTGTATCTGAGATGTGACTGTG
SAUR23_for	AT5G18060	ATGGCTTTGGTGAGAAGTCTATTGGT
SAUR23_rev	AT5G18060	TCAATGGAGCCGAGAAGTCACATTGA
GH3.5_for	AT4G27260	AGCCCTAACGAGACCATCCT
GH3.5_rev	AT4G27260	AAGCCATGGATGGTATGAGC
PIN1_for	AT1G73590	ATGGCTTCTGGTGGTGGTCGGAA
PIN1_rev	AT1G73590	AGCAGGACCACCGTCTTCTTCGT
PIN2_for	AT5G57090	TATCAACACTGCCTAACACG
PIN2_rev	AT5G57090	GAAGAGATCATTGATGAGGC
PIN3_for	AT1G70940	TGGTCCAAATCGTCGTCCTCCA
PIN3_rev	AT1G70940	TGGAAGCAGCCGTCTCAGGGA
PIN5_for	AT5G16530	CCATCGGCTCTATTGTCCTTG
PIN5_rev	AT5G16530	GCGACGAGCACAGGTAGAGA
